# Animal-friendly behavioral testing in field studies: examples from ground squirrels

**DOI:** 10.3389/fnbeh.2023.1239774

**Published:** 2023-08-23

**Authors:** Scott Nunes

**Affiliations:** Department of Biology, University of San Francisco, San Francisco, CA, United States

**Keywords:** animal-friendly, animal welfare, behavioral testing, field study, ground squirrel, rodent

## Abstract

Field studies of behavior provide insight into the expression of behavior in its natural ecological context and can serve as an important complement to behavioral studies conducted in the lab under controlled conditions. In addition to naturalistic observations, behavioral testing can be an important component of field studies of behavior. This mini review evaluates a sample of behavioral testing methods in field studies to identify ways in which behavioral testing can be animal-friendly and generate ethologically relevant data. Specific examples, primarily from studies of ground squirrels, are presented to illustrate ways in which principles of animal-friendly behavioral testing can be applied to and guide testing methods. Tests conducted with animals in their natural habitat and that elicit naturally occurring behavioral responses can minimize stress and disturbance for animals, as well as disruption of the larger ecosystem, and can have high ethological validity. When animals are trapped or handled as part of a study, behavioral testing can be incorporated into handling procedures to reduce overall disturbance. When behavior is evaluated in a testing arena, the arena can be designed to resemble natural conditions to increase the ethological relevance of the test. Efforts to minimize time spent in testing arenas can also reduce disturbance to animals. Adapting a behavioral test to a species or habitat conditions can facilitate reduced disruption to subjects and increased ethological relevance of the test.

## Introduction

Behavioral testing typically involves exposing an animal to a specific situation to assess a behavioral variable, and is an important component of neuroscience which can help elucidate elements of behavior under standardized conditions (Hernández-Arteaga and Ågmo, 2023). Laboratory studies are amenable to experimentally manipulating variables and conducting behavioral tests in controlled settings, and are important in establishing causal relationships between neural systems and expression of behavior. Field studies of behavior are less controlled, but allow for evaluation of behavior under naturalistic conditions in the context of the behavioral ecology of animals, and can serve as an important complement to laboratory studies (Nunes and Monroy Montemayor, 2023). In some cases, field studies provide information about behavior through basic observation of animals. For example, observation can provide information about motor skills associated with behavior and social interactions among individuals, as well as about how they vary among groups of individuals and change during development or across the lifespan (Meyer and Weber, 1996; Rho et al., 2007; Blumstein et al., 2013; Lee and Moss, 2014; Palagi, 2018; Gallo et al., 2021; Nolfo et al., 2021). Behavioral testing in a naturalistic field setting can reinforce observations, and in some cases provide a more feasible alternative to observation. For example, behavioral testing can be useful in the study of nocturnal or secretive animals whose behavior is difficult to directly observe, or in studies of rare events such as the threat of predation that might occur infrequently during regular observations (Tinbergen, 1948; Holekamp, 1986; Brehm et al., 2020). In developmental studies, behavioral testing can allow for finer-scale evaluation of behavior at specific time points or evaluation of behavioral changes across developmental periods. Moreover, behavioral testing can allow for data to be collected under uniform conditions, thereby controlling for possible variations in animals’ social or physical environments (Nunes and Monroy Montemayor, 2023).

Recently, d’Isa and Gerlai (2023) proposed guidelines for behavioral testing in lab settings that focus on the well-being of animals and relevance of the testing to the question being evaluated. They noted that minimizing stress during tests contributes to the ethical treatment of subjects, and also reduces possible confounding effects of stress on the outcome of tests. They further suggested that minimizing subjects’ contact with human handlers and designing tests that reflect the expression of behavior in naturally occurring contexts increase the reliability and replicability of tests, making results of tests more generalizable to settings beyond the lab. The guidelines proposed by d’Isa and Gerlai (2023) for animal-friendly behavioral testing in lab studies are also applicable to field studies. However, minimizing disruption to subject animals and the wider ecosystem are additional considerations in field studies. Trapping and handling methods, habitat features including anthropogenic alterations to the environment, and in some cases the presence of humans can generate physiological stress responses and influence behavior in free-living animals (Calsi and Bentley, 2009; Johnstone et al., 2012; Boonstra, 2013; Yardimci et al., 2013; Balestri et al., 2014; Huber et al., 2017; Boyle et al., 2021; Fardell et al., 2021). Benefits of field studies include evaluation of behavior in the context in which it naturally occurs and under which it evolved; however, behavioral testing that causes a high degree of disturbance to animals or their habitat can alter this context and negate the value of studying behavior in the field (Buchanan et al., 2012; Sikes et al., 2016).

Here I evaluate behavioral testing in field studies of free-living animals. Rodents are commonly used as model systems in lab and field studies of behavior. Ground squirrels in particular are amenable to behavioral studies in the field because they are diurnal, have relatively short life cycles (making developmental or longitudinal studies tractable), have relatively small home areas, typically occur at moderate to high population density within their habitats, and are fairly easy to handle (Wolff and Sherman, 2007). I assess behavioral testing methods in the context of their friendliness and ethological relevance to subject animals and provide some specific examples, primarily from studies of ground squirrels. I focus on basic tenets of animal-friendly testing including (1) minimizing stress to subject animals, (2) reducing disturbances to subject animals and their habitat, (3) creating standardized conditions for tests, and (4) developing tests germane to the ethology and behavioral ecology of animals. The goal here is to illustrate basic ways that these principles can be applied to and guide behavioral testing of free-living animals.

## Motor skill and development

Field studies of motor development have helped elucidate various features of behavior, including development of anti-predator behavior, benefits of juvenile play, the timing of natal dispersal, and energetic costs of behavioral development (Nunes et al., 2004; Berghänel et al., 2015; Carter et al., 2019; Gallo et al., 2021). Development of motor and executive areas of the brain extends into the juvenile period in a wide range of animals (Watson et al., 2006; Stiles and Jernigan, 2010; White and Sillitoe, 2013; Sakai and Sugiyama, 2018), and field studies of motor development can help to identify possible periods of motor and behavioral development in the brain of species not commonly studied in the lab (Carter et al., 2019). In studies of larger animals or animals with relatively long periods of juvenile development, evaluation of motor function and motor development typically involves longitudinal observation or videotaping of motor skills displayed during regular activity, to monitor performance of behavior and improvement in motor skill and coordination over time (Berghänel et al., 2015; Carter et al., 2019; Gallo et al., 2021).

**FIGURE 1:**
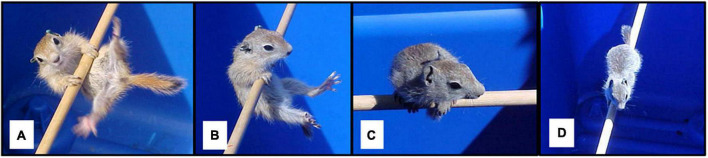
Motor skill test for Belding’s ground squirrels. Squirrels were placed on a cylindrical wooden rod **(A)**, and their responses were observed. Squirrels could immediately fall, hang on rod **(B)**, climb onto the rod and perch with the body perpendicular to the rod **(C)**, balance on the rod with body perpendicular to the rod **(D)**, walk along the rod, or jump from the rod to the edge of the arena. Squirrels were given scores based on the final outcome of the test, with scores increasing with the difficulty of skills needed to achieve an outcome. Tests were terminated when the squirrel fell off the rod, jumped to the rim of the arena, or after 1 min, whichever came first. Adapted from Nunes et al. (2004).

Behavioral testing to evaluate motor skill might not provide the same ecological context as naturalistic observations, but can allow for assessment of motor skill on a finer scale and with greater standardization of conditions than basic observations. For example, Nunes et al. (2004) evaluated development of motor skill in juvenile Belding’s ground squirrels (*Urocitellus beldingi*) with tests that required increasingly skilled behavior to progress through the task (Figure 1). Squirrels in this species have a relatively short period of juvenile development and because of their small size can be collected and handled with relatively simple and quick procedures, helping to minimize overall disruption to the animals. Testing that involved progression through different skill levels revealed emergence of new skills and increased motor proficiency at different points of development, and controlled for the possibility that the testing procedure itself provided practice and promoted development of specific skills. To mitigate disruptions associated with testing, tests were conducted in squirrels’ home areas, which avoided transporting squirrels. Squirrels were tested immediately after being collected and were released immediately after tests were completed, to minimize the time they were removed from their home environment.

## Alarm calls

Many species across a range of taxa use alarm calls to communicate information about predators or other potential threats (Slobodchikoff, 2010; Gill and Bierema, 2013; Townsend and Manser, 2013). Within a species, animals can vary alarm vocalizations to encode specific information such as the degree or imminence of danger posed by a predator or potential threat (Zuberbühler et al., 1997; Zuberbühler, 2000; Murphy et al., 2013; Coye et al., 2015; Carlson et al., 2017). Because alarm calls communicate information about possible danger, they can elicit specific vigilant or antipredator behavioral responses, as well as physiological responses, in conspecifics who hear the calls (Mateo, 2010; Silvestri et al., 2019; McRae, 2020; Lawson et al., 2021). Evaluation of alarm calls during trapping procedures can provide information about the health status of yellow-bellied marmots (*Marmota flaviventer*; Nash et al., 2020). Moreover, playing recordings of alarm vocalizations can serve as a minimally disruptive testing method for evaluating various elements of vigilant or antipredator behavior. Recordings present stimuli that animals encounter during regular activity, and evoke responses germane to the behavioral ecology of animals. For example, playback of alarm calls have been an important component of behavioral testing in studies assessing variation among individuals in antipredator behavior, the influence of social relationships on perceptions of threat and safety, and responsiveness to communication and signaling from different species or different populations of the same species (Aschemeier and Maher, 2011; Lea and Blumstein, 2011; Makenbeach et al., 2013; Blumstein et al., 2017; Lengagne et al., 2020).

Studies of ground squirrels involving playback of alarm calls have also evaluated the trade-off between body condition and vigilance. Arenz and Leger (2000) supplemented some juvenile thirteen lined ground squirrels (*Ictidomys tridecenlineatus*) with high energy food to manipulate body mass and body condition. They observed the vigilant and foraging behavior of juveniles, and found that unsupplemented juveniles foraged more and displayed less vigilant behavior than did supplemented juveniles. Bachman (1993) similarly manipulated body condition of adult and yearling female Belding’s ground squirrels by supplementing some squirrels with high energy food. She later set up behavioral testing stations with high energy food, and played recordings of alarm calls to assess vigilant responses when squirrels came to feed. Unprovisioned squirrels expressed less vigilant behavior and were more likely to continue feeding when alarm calls were played. These two studies took different approaches to evaluate similar research questions, but their approaches acted synergistically to increase the reliability of the finding that animals may reduce vigilance in favor of foraging when they have smaller energy reserves. Behavioral testing provided evaluation of behavior under relatively uniform conditions, whereas naturalistic observations demonstrated a tradeoff between vigilance and foraging in the daily activity of individuals.

## Temperament

Expression of behavior varies among individuals, and behavioral traits of individuals that show consistency over time and across situations are generally referred to as temperament. Elements of temperament comprise behaviors that vary along continua. For example, the caution-boldness continuum includes responses to risks or threats, the avoidance-exploration continuum includes responses to novel objects or situations, and the docility continuum includes the degree to which responses in a situation are passive vs. active (Sih et al., 2004; Réale et al., 2007, 2010; Herde and Eccard, 2013; Petelle et al., 2013). Evaluation of temperament has a range of important applications to the study of human mental health, neural correlates of behavior, physiological responses to stress, the welfare of captive animals, social behavior and social interaction, antipredator behavior, space use, dispersal, behavioral development, and an array of ecological variables (Carere et al., 2001; Dingemanse et al., 2004; Both et al., 2005; Boon et al., 2008; Clary et al., 2014; Vetter et al., 2016; Rasmussen and Belk, 2017; Hecht et al., 2021; MacGregor et al., 2021; Pomerantz and Capitanio, 2021; Wauters et al., 2021; Skinner et al., 2022; Luciano et al., 2023; Nunes and Monroy Montemayor, 2023). Here I discuss behavioral testing methods related to assessing elements of temperament, and provide examples of methods used to evaluate development of temperament along the caution-boldness and docility continua in Belding’s ground squirrels.

Flight-initiation distance tests (henceforth flight tests) gauge the distance at which an individual flees from an approaching human and are commonly used to evaluate temperament along the caution-boldness continuum (Ydenberg and Dill, 1986; Blumstein, 2003; Runyan and Blumstein, 2004). Flight is an antipredator response, and flight tests are considered to provide a measure of caution or boldness in response to a threat (Cooper, 2009; Petelle et al., 2013). Flight tests elicit a response among subjects, but do not require trapping or handling, minimizing stress to subject animals and disturbance to the local habitat. Flight tests have been an integral component of a range of studies addressing diverse research questions related to energetic influences on behavior, behavioral strategies in reproduction, behavioral adaptations to local environmental conditions, species distributions based on interactions between behavior and habitat, and behavioral responses to climate change (Shuai et al., 2019, 2022; Pereira et al., 2020; Satterfeld and Johnson, 2020; Stamoulis et al., 2020; Díaz et al., 2021; Hamao et al., 2021; Ventura et al., 2021; Mikula et al., 2023).

The ethological relevance of flight tests can vary. Some species do not distinguish between human intruders and natural predators, and flight distances during tests do not differ when individuals are approached by a human compared to a predator (e.g., Asunsolo-Rivera et al., 2023). However, other species have nuanced responses to threats and discriminate between different levels of threat or different types of predators, and flight distances in response to human intruders can differ from those in response to actual predators (Allan et al., 2021; Morelli et al., 2022). Thus, in studies specifically evaluating antipredator behavior, rather than temperament in general, behavioral observations of responses to predators would increase the reliability of results obtained from flight tests.

Prior interactions with people and levels of local human activity can influence the outcomes of flight tests. During repeated trials over a short time period, test subjects can become habituated to human intruders and flee at shorter approach distances (Petelle et al., 2013). Similarly, in areas with high human population density, animals can become acclimated to people and flee at shorter distances during flight tests (Ekanayake et al., 2022). In some cases, influences of human activity on results of flight tests can be applied to understanding human-wildlife coexistence and can provide insights into behavioral responses to environmental changes caused by anthropogenic activity (Pettit et al., 2021; Mikula et al., 2023).

**FIGURE 2:**
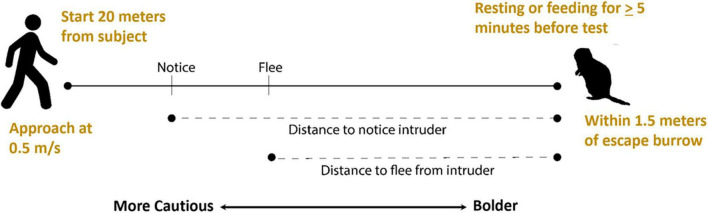
Flight initiation distance (flight) tests for Belding’s ground squirrels. A human intruder identifies a subject who has been feeding or resting continuously for at least 5 min, starts at a set distance from the squirrel, walks at a constant rate toward the squirrel, and marks the distances at which the squirrel notices and flees from the intruder with greater distances reflecting greater caution.

Because flight tests do not involve trapping or handling animals and mimic disturbances individuals might encounter during regular activity, they can be useful in evaluating behavioral development without introducing variables that could potentially influence developmental processes. Shehan et al. (2023) developed a flight test to assess a possible association between play behavior and the development of cautious responses in juvenile Belding’s ground squirrels (Figure 2). They evaluated distances at which juvenile squirrels first noticed and then fled from a human intruder, with greater distances reflecting greater caution. They observed that caution increased as juveniles got older and increases were positively correlated with rates of social play, raising the possibility that play behavior may have a role in development of cautious responses in young squirrels.

Ramos et al. (2023) noted that individual responses to trapping or handling can provide information about temperament and suggested that disturbances to animals can be reduced by incorporating assessment of temperament into regular data collection procedures that involve trapping and handling. Evaluation of docility in particular is amenable to being integrated into handling methods. For example, Kannan et al. (2022) used passive vs. active responses of captive goats (*Caprus hircus*) while being weighed as a measure of excitability. Petelle et al. (2013) used passive vs. active responses of free-living yellow-bellied marmots while in traps as a measure of docility. Underhill et al. (2021) evaluated docility in free-living mice (*Peromyscus leucopus* and *P. maniculatus*) and DeRango et al. (2019, 2021) evaluated docility in free-living Galápagos sea lions (*Zalophus wollebaeki*) as the degree to which individuals struggled while being handled. Measurements of docility during handling and trapping have limits in that they do not directly reflect behaviors expressed during regular activity in animals’ natural habitat. However, they are generally considered to represent tendencies toward reactive or proactive behaviors not related to threats or novelty, and have been important in studies of behavioral and physiological stress responses, behavioral plasticity, behavioral development, stability of individual behavior across the lifespan, and the degree to which behavioral traits can predict other features of behavior (Réale et al., 2000, 2009; Petelle et al., 2013, 2015, 2017; DeRango et al., 2019, 2021; Underhill et al., 2021; Kannan et al., 2022).

**FIGURE 3:**
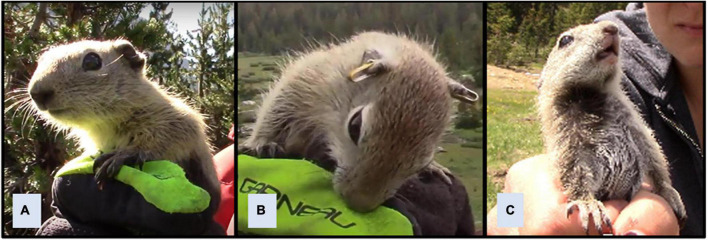
Docility tests for Belding’s ground squirrels. Squirrels are held and their responses are recorded for 30 s. Responses such as remaining still **(A)** are scored as passive, and responses such as biting the handler’s glove **(B)** or struggling to escape **(C)** are recorded as active. Docility scores are calculated as the number of seconds during tests that juveniles are passive. Adapted from Hurst-Hopf et al. (2023).

Hurst-Hopf et al. (2023) evaluated the relationship between play behavior and the development of temperament along the docility continuum in Belding’s ground squirrels. Docility tests were incorporated into handling procedures, and consisted of holding juvenile squirrels and videotaping their responses for 30 s (Figure 3). Responses shifted to being less passive and more active as juveniles got older. This shift was correlated with rates of social play, raising the possibility that play behavior may refine development of temperament in young squirrels. Responses during docility tests were not directly generalizable to specific behaviors within the behavioral repertoires of squirrels, but contributed to formulation of a developmental hypothesis suggesting that as juvenile squirrels venture farther from the natal burrow, behavioral responses become more proactive to facilitate gathering of information about the social and physical environment, while cautious responses increase to reduce vulnerability to predation (Nunes and Monroy Montemayor, 2023).

## Remote monitoring

Technologies that allow for monitoring animals remotely without the presence of people can reduce disruption to animals and their habitats and eliminate confounding effects that may be associated with human observers nearby (Trathan and Emmerson, 2014). Radio-frequency identification (RFID) systems have important applications for remote monitoring in behavioral testing in free-living rodents as well as a range of other vertebrates (Dell’Omo et al., 1998; Ousterhout and Semlitsch, 2014; Fetherman et al., 2017; Hughes et al., 2021; Stryjek et al., 2021; Harrison and Kelly, 2022). In RFID systems, a small passive-integrated transponder (PIT) tag is implanted subcutaneously using a minimally-invasive procedure. The PIT tag facilitates lifetime identification of an individual without external tagging or marking. Antennas can be set up to read PIT tags and record the presence or movement of animals at burrow entrances or nesting sites, natural foraging patches, experimental feeding stations, or established runways regularly used by animals (Dell’Omo et al., 1998). Remote monitoring with RIFD technology can have important applications in a range of studies of free-living rodents including evaluation of exploratory behavior, risk perception and aversion, structure of social grouping, environmental effects on social affiliation and activity patterns, and effects of social connection on disease transmission and immune system responses (Perony et al., 2012; Schuett et al., 2012; Scheibler et al., 2013, 2014; Halliday et al., 2014; König et al., 2015; Lopes et al., 2016, 2020; Bleicher et al., 2018; He et al., 2019; Evans et al., 2021).

## Heuristic approaches to design new animal-friendly behavioral tests

Finding ways to adapt behavioral tests to a specific research question, species, or habitat conditions can increase the ethological and ecological relevance of a study and reduce disruption to subjects. For example, Drayton and Santos (2017) evaluated the degree to which non-human animals are aware of what other individuals know. They worked with a population of rhesus macaques (*Macaca mulatta*) on the island of Cayo Santiago in Puerto Rico where macaques are accustomed to the presence of humans. They set up a testing station with behavioral tests that involved macaques following the gaze of a human, and conducted tests when macaques entered the testing area on their own. Drayton and Santos (2017) considered specific features of the population from which subjects were drawn, taking advantage of the macaques’ freedom to roam across the island and familiarity with humans to design an animal-friendly behavioral test that did not involve handling macaques or interfering with their regular activity. Moreover, they made use of a behavioral response (gaze-following) present in the animals’ natural behavioral repertoire. Macaques followed the gaze of a human observing an object, and the macaques’ gaze-following varied with how familiar the human was with the object, suggesting that macaques are cognizant of what other individuals know.

**FIGURE 4:**
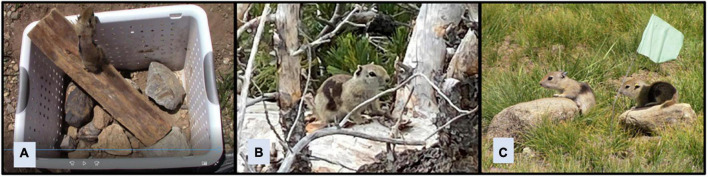
Problem-solving test for Belding’s ground squirrels. A squirrel is placed in a testing arena, and the amount of time needed for the squirrel to escape is recorded **(A)**. Methods of escape include using objects from the squirrel’s natural environment such as branches **(B)** and rocks **(C)**.

Marks et al. (2017) evaluated the relationship between play behavior in juvenile Belding’s ground squirrels and development of the ability to navigate novel situations. They designed a behavioral test that involved placing a juvenile squirrel in an unfamiliar testing arena and recording the amount of time the squirrel needed to escape from the arena. Although the test was conducted in an arena rather than the squirrels’ natural habitat, attempts were made to have the arena mimic the natural habitat by equipping it with objects that squirrels encounter in their habitat during regular activity, such as branches and rocks, that could be used as an aid to escape from the arena (Figure 4). Tests were terminated after 1 min if squirrels had not escaped by then, to minimize disturbance to squirrels. In addition to minimizing disturbance, limiting the amount of time subjects spend in a testing arena and the number of times they are placed in the arena reduce the likelihood that they will become familiar with the arena or acclimated to testing procedures, which could affect the outcomes of tests conducted in the arena in the future (Ozawa et al., 2011). The time that juvenile squirrels took to escape from the testing arena was found to be associated with their play behavior, suggesting that play might help prepare young animals to navigate unfamiliar situations.

## Conclusion

Naturalistic observations and behavioral testing can importantly complement each other in field studies. Observations place results in the context of animals’ behavioral ecology, and behavioral testing allows for evaluation of behavior under standardized conditions. Animal-friendly tests that are minimally disruptive not only benefit the welfare of animals but also generate ethologically relevant results. Animal-friendly tests can use a variety of approaches to increase their ethological and ecological relevance to the research question or animals being studied. Tests conducted with subjects in their natural habitat ideally involve eliciting behaviors expressed by the animals during regular activity. When animals are trapped or handled in a study, behavioral tests can be designed to evaluate responses to handling, thereby maximizing data collection during handling and eliminating the need for separate testing. When behavior is evaluated in a testing arena, arranging the arena to resemble natural conditions can support the ethological relevance of the test, and minimizing time spent in the arena can reduce disruption to subjects. Taking into account the behavior and ecology of a species when designing or adapting a behavioral test for free-living animals can help to maximize the overall relevance of the test.

## Author contributions

SN conceived, wrote, and edited this mini review.
